# Iatrogenic Dysnatremias in Children with Acute Gastroenteritis in High-Income Countries: A Systematic Review

**DOI:** 10.3389/fped.2017.00210

**Published:** 2017-10-06

**Authors:** Silviu Grisaru, Jianling Xie, Susan Samuel, Stephen B. Freedman

**Affiliations:** ^1^Section of Pediatric Nephrology, Alberta Children’s Hospital, Alberta Children’s Hospital Research Institute, Cumming School of Medicine, University of Calgary, Calgary, AB, Canada; ^2^Section of Pediatric Emergency Medicine, Alberta Children’s Hospital, Cumming School of Medicine, University of Calgary, Calgary, AB, Canada; ^3^Section of Emergency Medicine, Department of Pediatrics, Alberta Children’s Hospital, Alberta Children’s Hospital Research Institute, Cumming School of Medicine, University of Calgary, Calgary, AB, Canada; ^4^Section of Gastroenterology, Department of Pediatrics, Alberta Children’s Hospital, Alberta Children’s Hospital Research Institute, Cumming School of Medicine, University of Calgary, Calgary, AB, Canada

**Keywords:** hyponatremia, intravenous fluids, diarrhea, children, gastroenteritis, isotonic solutions

## Abstract

**Background:**

Acute gastroenteritis (AGE) causing dehydration with or without dysnatremias is a common childhood health challenge. While it is accepted that oral rehydration therapy is preferred, clinical factors or parent and healthcare provider preferences may lead to intravenous rehydration (IVR). Isotonic solutions are increasingly recommended in most scenarios requiring IVR. Nevertheless, children with AGE, having ongoing losses of water and electrolytes, represent a unique population.

**Objectives:**

To evaluate the association between acquired dysnatremias and IVR in children with AGE.

**Methods:**

A systematic search of MEDLINE database was conducted through September 14, 2016. Observational studies and clinical trials conducted in high-income countries were included. The Grades of Recommendation, Assessment, Development, and Evaluation approach was used to evaluate the overall quality of evidence for each outcome.

**Results:**

603 papers were identified of which 6 were included (3 randomized controlled trials and 3 observational studies). Pooling of patient data was not possible due to significantly different interventions or exposures. Single studies results demonstrated that within 24 h, administration of isotonic saline was not associated with a significant decline in serum sodium while hypotonic solutions (0.2–0.45% saline) were associated, in one study, with mean serum sodium declines from 1.3 mEq/L (139.2, SD 2.9–137.9, SD 2.5) in 133 young infants (aged 1–28 months), to 5.7 (SD 3.1) mEq/L in a subgroup of 18 older children (age mean 5.8, SD 2.7 years). Both isotonic and hypotonic saline were shown to be associated with improvement of baseline hyponatremia in different studies. Baseline hypernatremia was corrected within 4–24 h in 81/83 (99.6%) children using hypotonic saline IVR.

**Conclusion:**

There is a paucity of publications assessing the risk for acquired dysnatremias associated with IVR in children with AGE. Current high-quality evidence suggests that, short-term use of isotonic solutions is safe and effective in most children with AGE; hypotonic solutions may also be appropriate in some subpopulations, however, the quality of available evidence is low to very low. Further research investigating outcomes associated with IVR use beyond 24 h focusing on specific age groups is required.

## Background

Dehydration, complicating acute gastroenteritis (AGE), continues to be a leading cause of morbidity and mortality around the globe (1). Numerous studies have confirmed the safety and efficacy of oral rehydration therapy (ORT), thus it is considered the preferred method of rehydration in children with AGE (2). Nevertheless, intravenous rehydration (IVR) continues to be frequently employed in settings where ORT is clinically inappropriate (i.e., obtunded child, intractable vomiting, and severe dehydration), and at times when it may not be necessary but it is deemed the preferable therapy by families or physicians (3, 4).

Both oral and IVR can cause or exacerbate electrolyte disturbances related to sodium homeostasis. The risk for hypernatremia associated with oral rehydration solutions (ORSs) containing excessive amounts of salt and carbohydrates was dramatically reduced by the introduction of reduced total osmolality ORS containing equimolar concentrations of sodium and glucose (5–7). While the risk of hyponatremia exists with the use of ORT, it is exceedingly minimal in children with none to minimal dehydration, as reflected in a recent study that allowed children to drink preferred alternatives to standard ORS (8). However, hospitalized children have a greater risk of developing hyponatremia due to the presence of excessive antidiuretic hormone (ADH) that limits the body’s ability to excrete water. This potential may be exacerbated when hypotonic saline solutions are administered intravenously to maintain hydration (9). While the use of isotonic saline solutions may mitigate the risk of hyponatremia in children with excessive ADH secretion (10, 11), concerns related to sodium and fluid overload exist. These concerns provide the basis for the current debate regarding the optimal IVR solution composition in children (12, 13). The topic is of particular importance in children hospitalized with dehydration secondary to AGE, as such children may present with dysnatremias and frequently have substantial ongoing loses of water and electrolytes (14).

To form an evidence-based opinion on this topic, we conducted a systematic review of clinical trials and observational studies that investigated the risk of dysnatremias in relation to IVR solution composition and rate of administration. We focused on children in high-income countries given the significant differences in patients, pathogens, and management practices that exist, driven by economic factors.

## Methods

This review was planned, conducted, and reported in adherence with Preferred Reporting Items for Systematic Reviews and Meta-Analyses standards of quality for reporting systematic review (15).

### Patient/Population, Intervention, Comparator, Outcome (PICO) Question

We sought to identify studies to address the following PICO question (16): in children with AGE, who receive intravenous fluid (IVF), in any setting, in high-income countries (P), are different types of fluid (i.e., isotonic and hypotonic), different infusion rates, total volumes, or patient baseline characteristics (I, C) associated with different risks of developing dysnatremia (O)?

### Study Eligibility

Eligible studies contained the following elements: (1) children with AGE; (2) all patients received IVF therapy; (3) compared different types of IVF, or different infusion rates or different IVF volumes, or investigated risk factors in patient characteristics (i.e., baseline dysnatremia caused by dehydration); (4) clinical or biochemical documentation of hydration status, fluids administered, and serum sodium level before and after fluid therapy; (5) conducted in a high-income country (http://data.worldbank.org/income-level/high-income); and (6) with any outcome measure of serum sodium after IVR treatment. All eligible studies, regardless of report focus, were included. Review articles, and those containing duplicate data were excluded. No language, age, or study design restrictions were employed.

### Data Source and Search

We searched MEDLINE (1946 to September 2016) on September 14, 2016. The search used key terms including Gastroenteritis, Diarrh*ea, Vomiting, Volume expansion, Fluid management, IVF, Hydration, Dehydration, Intravascular volume expansion, Fluid, Saline, Rehydration, Hyponatr*emia, and Hypernatr*emia. The MEDLINE search strategy is appended (see Table S1 in Supplementary Material). Experts in the field were also invited to suggest relevant studies. All published studies with sufficient information, irrespective of language of publication, publication year, publication type, and publication status, were eligible for inclusion. An updated search performed on May 5, 2017 identified no additional eligible studies.

### Study Selection

Potentially relevant publications were identified through independent screening of search result titles and abstracts by two trained study reviewers that included a pediatric emergency medicine physician (Jianling Xie) and a pediatric nephrologist (Silviu Grisaru). The reviewers had an overall agreement of 97.6%. Duplicate articles were manually reconciled.

The full text of potentially relevant citations was obtained and then reviewed by two independent reviewers (Silviu Grisaru and Jianling Xie) using standard, predefined eligibility criteria. A consensus approach with input from the senior investigator (Stephen B. Freedman) was employed to resolve disagreements. Reasons for inclusion and exclusion were documented.

### Data Collection and Analysis

Data were extracted by two independent reviewers (Jianling Xie and Silviu Grisaru) and any disagreement was resolved by discussion to reach consensus. We collected information on the study design, research topic, nature of participant characteristics, IVF type, infusion rate and volume, follow-up interval, and outcomes type and outcome data. We only collected data published, unpublished data were not sought. We did not perform meta-analysis as all included studies investigated different interventions or exposures that precluded data synthesis, thus only a narrative summary of results was generated.

### Risk of Bias Assessment and Grades of Recommendation, Assessment, Development, and Evaluation (GRADE) Approach

The Newcastle–Ottawa scale (NOS) (17) was used to assess the risk of bias for observational studies in three domains: (1) cohort selection; (2) comparability; and (3) outcome assessment using eight multiple choice questions. A study is deemed to be of good, fair, or poor quality if the score is ≥7, 6, and ≤5 (out of 9), respectively (18). The Cochrane Risk of Bias tool was used to assess randomized trials with five different domains of quality (19): (1) randomization generation; (2) allocation concealment; (3) blinding; (4) complete accounting of patients and outcome event; and (5) outcome reporting. The GRADE approach (19, 20) classifies the quality of evidence into four categories (high, moderate, low, and very low), according to five domains of the studies (1) risk of bias; (2) inconsistency; (3) indirectness; (4) imprecision; and (5) publication bias (19). Assessment was conducted by one author (Jianling Xie) and verified by a second author (Silviu Grisaru).

## Results

### Study Selection

**Figure 1:**
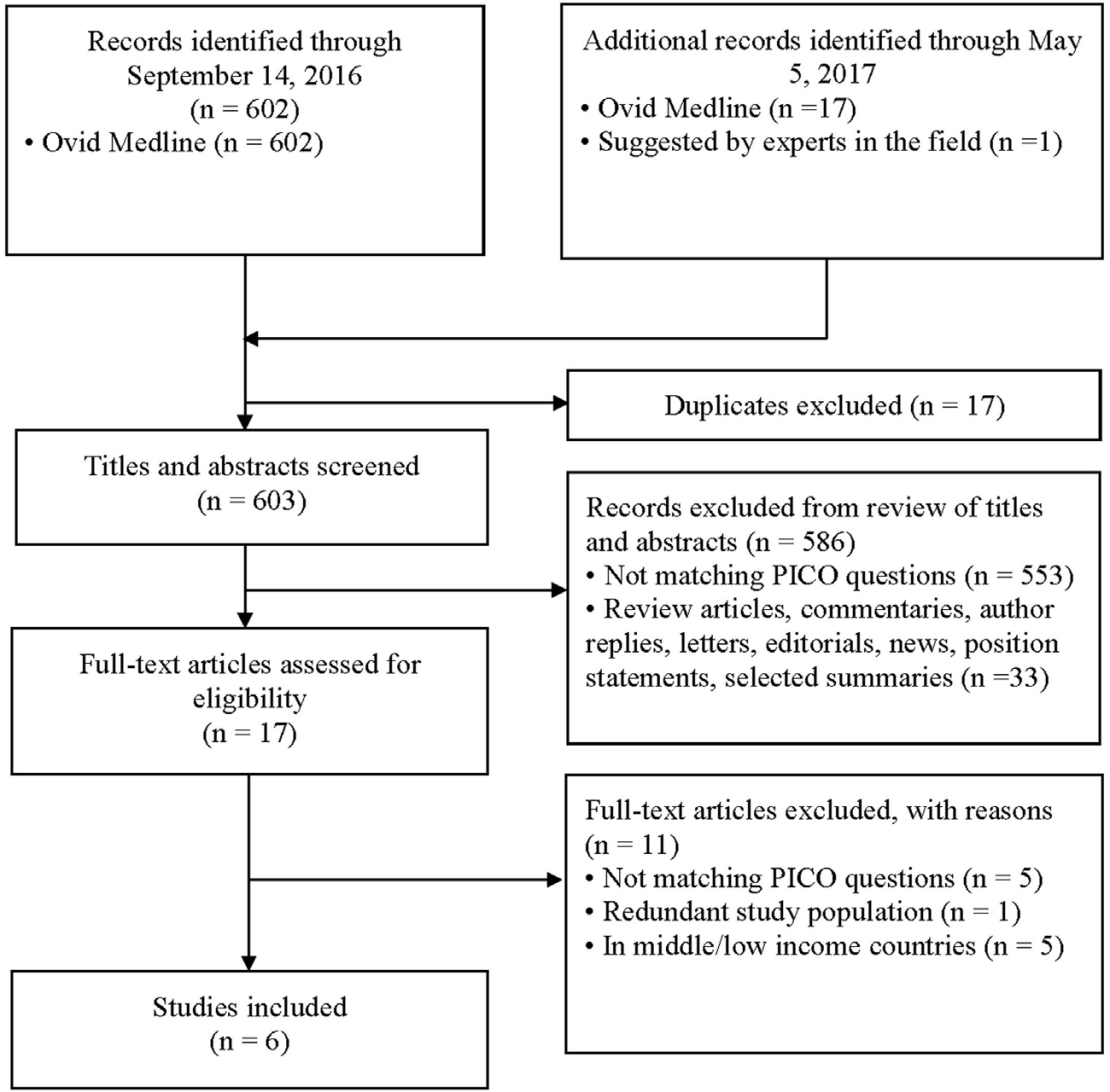
Selection of studies for inclusion in the systematic review.

Six hundred and two abstracts were identified by the initial search and one additional study was suggested by an expert in the field; at the end of the screening process and application of exclusion/inclusion criteria, six eligible studies were included in this review (Figure 1). The eligible studies included three randomized controlled trials (21–23); two prospective observational studies (24, 25); and one retrospective chart review (26). A brief overview of the included studies is shown in Table 1. All included studies demonstrated that IVR-related serum sodium outcomes were strongly influenced by baseline serum sodium values suggesting that grouping patients and outcomes according to baseline serum sodium status (i.e., hyponatremia, isonatremia, and hypernatremia) is a logical approach (Tables 2–4).

**Table 1:** Overview of included studies.

Reference	Country	Age, years, mean (SD)	Design	Study population	IVR solutions	Time to repeat serum sodium
Neville et al. (22)	Australia	2.9 (2.0)	RCT	*n* = 102, age 6 months to 14 years, AGE with dehydration, dehydration score 3–7	IVR D2.5-0.9% saline (*n* = 51) vs. D2.5-0.45% saline (*n* = 51), rate: either RRP or SRP	4 h

Freedman and Geary (21)	Canada	2.9 (2.1)	RCT	*n* = 224, children >90 days, AGE with dehydration, clinical dehydration score >3	IVR 0.9% saline, 60 mL/kg (*n* = 114) vs. 20 mL/kg (*n* = 110), rate: over an hour, followed by D5-0.9% saline at maintenance rate	4 h

Allen et al. (23)	USA and Canada	2.9–3.8 (NA)	RCT	*n* = 77, age 6 months to 11 years, AGE with dehydration, Gorelick dehydration score (29) ≥4	IVR 0.9% saline vs. Plasma-Lyte A, 10–20 mL/kg allotments until clinical rehydration or up to 8 h	4 h

Sánchez-Bayle et al. (25)	Spain	0.96 (0.48)	Prospective observational	*n* = 205, age 1–28 months, AGE with mild to moderate dehydration	IVR D5-0.3% saline (*n* = 198), IVR D5-0.2% saline (*n* = 7), rate: mean, 5.51 (SD 1.3) mL/kg/h	At an average 12.34 h (95% CI: 11.94, 12.56)

Kahn et al. (24)	Belgium	0.26 (0.02)	Prospective observational	*n* = 40, infant <6 months, AGE with severe hypernatremic dehydration sodium ≥155 mEq/L	~70 mEq/L sodium (1:1 mixture of D5 with145 mEq/L saline), rate: 120 mL/kg/24 h	Every 6 h for 24 h

Hanna and Saberi (26)	USA	3.3 (3.1)	Retrospective chart review	*n* = 124, age 1 months to 12 year, AGE with moderate to severe dehydration, baseline sodium 130–150 mEq/L	IVR D5-0.2% saline (*n* = 4), IVR D5-0.3% saline (*n* = 102), IVR D5-0.45% saline (*n* = 18), rate: maintenance + deficit over 24 h	Within 24 h (mean 13.2, SD 5.2)

*IVR, intravenous rehydration; AGE, acute gastroenteritis; RCT, randomized control trial; D5, 5% dextrose water; D2.5, 2.5% dextrose water; RRP, 10 mL/kg/h for 4 h; SRP, maintenance fluids + estimated dehydration as a percentage of body weight replaced over 24 h; N/A, not available*.

**Table 2:** Summary of results in patients with hyponatremia at baseline.

Reference	IVR solution	Volume administered mean (SD)	Baseline hyponatremia^a^ *n* (%)	ΔNa (moll/L), mean (SD)	Hyponatremia at follow-up *n* (%)^b^	Hypernatremia at follow-up *n* (%)^b^
Neville et al. (31)	D2.5-0.45% saline	RRP or SRP for 4 h	16/102 (15.7)	0.4 (1.7)	N/A	0
	D2.5-0.9% saline		21/102 (20.6)	2.4 (1.5)	N/A	0

Freedman and Geary (21)	0.9% saline	60 mL/kg/h bolus + maintenance for 3 h	48/224 (21.4)	2.9 (1.9)	18/48 (37.5)	0
		20 mL/kg/h bolus + maintenance for 3 h	36/224 (16.1)	2.4 (2.2)	19/34 (56)	0

Allen et al. (23)	0.9% saline	10–20 mL/kg boluses until rehydrated for up to 8 h	8/38 (21.1)	N/A	4/8 (50)	
	Plasma-Lyte A		13/39 (33.3)	N/A	8/13 (61.5)	

Sánchez-Bayle et al. (25)	D5-0.3% saline	5.51 (1.3) mL/kg/h for 12.34 h (95% CI: 11.94, 12.56)	37/205 (18.0)	3.7 (N/A)	0	0

Hanna and Saberi (26)	0.9% saline bolus + D5-0.2–0.45% saline	Maintenance + deficit/24 h for 13.2 (5.2) h	19/124 (15.3)	3.9 (2.5)	5/19 (26.3)	0

*D5, 5% dextrose water; D2.5, 2.5% dextrose water; N/A, not available; RRP, 10 mL/kg/h for 4 h; SRP, maintenance fluids + estimated dehydration as a percentage of body weight replaced over 24 h*.

*^a^Definition for hyponatremia, Freedman and Geary (21), serum sodium <136 mEq/L; Hanna and Saberi (26), ≤134 mEq/L; Neville et al. (31) and Sánchez-Bayle et al. (25), <135 mmol/L; Allen et al. (23), serum sodium <135 mEq/L*.

*^b^Time when this assessment was done after IVR: in Freedman and Geary (21), Neville et al. (31) studies, and Allen et al. (23), at 4 h; in Henna and Saberi (26) study, within 24 (mean 13.2, SD 5.2) h; in Sánchez-Bayle et al. (25) study, in 12.34 h (95% CI: 11.94, 12.56)*.

**Table 3:** Summary of results in patients with isonatremia (normonatremia) at baseline.

Reference	IVR solution	Age, years, mean (SD)	Volume administered mean (SD)	Baseline isonatremia^b^ *n* (%)	ΔNa (mmol/L), mean (SD)	Hyponatremia at follow-up *n* (%)^a,c^	Hypernatremia at follow-up *n* (%)^a^
Neville et al. (31)	D2.5-0.45% saline	3.1 (2.0)	RRP or SRP	35/102 (34.3)	−2.3 (2.2)	N/A	0
	D2.5-0.9% saline	2.7 (1.5)		30/102 (29.4)	0.8 (2.4)	N/A	0

Freedman and Geary (21)	0.9% saline	2.9 (2.1)	60 mL/kg bolus + 3 h maintenance	63/224 (28.1)	0 (N/A)	1/63 (1.6)	0
		3.0 (2.2)	20 mL/kg bolus + 3 h maintenance	73/224 (32.5)		0	0

Allen et al. (23)	0.9% saline	2.9 (N/A)	39.6 mL/kg boluses + 12.3 mL/kg maintenance × 1.6 h	30/38 (79)	N/A	1/30 (3.3)^c^	0
	Plasma-Lyte A	3.8 (N/A)	38.4/kg boluses + 12.2 mL/kg maintenance × 1.7 h	26/39 (67)	N/A	1/26 (3.9)^c^	0

Sánchez-Bayle et al. (25)	D5-0.3% saline	N/A	5.51 (1.3) mL/kg/h	133/205 (64.8)	−1.26 (N/A)	0	0

Hanna and Saberi (26)	0.9% saline bolus + D5-0.2–0.45% saline	2.8 (3.1)	4.8 (1.6) mL/kg/h	79/124 (63.7)	−1.8 (3.4)	0	0
		5.8 (2.7)	4.3 (1.6) mL/kg/h	18/124 (14.5)	−5.7 (3.1)	18/18 (100%)	0

*D5, 5% dextrose water; D2.5, 2.5% dextrose water; N/A, not available; RRP, 10 mL/kg/h for 4 h; SRP, maintenance fluids + estimated dehydration as a percentage of body weight replaced over 24 h*.

*^a^Time when this assessment was done after IVR: in Freedman and Geary (21), Neville et al. (31), and Allen et al. (23) studies, at 4 h; in Hanna and Saberi (26) study, within 24 (mean 13.2, SD 5.2) h; in Sánchez-Bayle et al. (25) study, in 12.34 h (95% CI: 11.94, 12.56)*.

*^b^Definition for isonatremia, Freedman and Geary (21), serum sodium 136–145 mEq/L; Allen et al. (23), Hanna and Saberi (26), Neville et al. (31), and Sánchez-Bayle et al. (25), serum sodium 135–145 mEq/L*.

*^c^Definition of hyponatremia, Freedman and Geary (21), serum sodium <136 mEq/L; Allen et al. (23), serum sodium <135 mEq/L*.

**Table 4:** Summary of results in patients with hypernatremia at baseline.

Reference	IVR solution	Volume administered mean (SD)	Baseline hypernatremia^a^ *n* (%)	ΔNa (mmol/L), mean (SD)	Hyponatremia at follow-up *n* (%)^b^	Hypernatremia at follow-up *n* (%)^b^
Sánchez-Bayle et al. (25)	D5-0.2–0.3% saline	5.51 (1.3) mL/kg/h	35/205 (17.1)	−8.15 (N/A)	0	0
Kahn et al. (24)	D5- Na 70 mEq/L	120 mL/kg/24 h	40/40 (100)	−0.265 (0.036) mmol/L/h	N/A	N/A
Hanna and Saberi (26)	0.9% saline bolus + D5-0.2–0.45% saline	Maintenance + deficit	8/124 (6.5)	−5.9 (3.8)	0	2/8 (25%)

*D5, 5% dextrose water; N/A, not available*.

*^a^Definition for hypernatremia, Freedman and Geary (21), Hanna and Saberi (26), and Sánchez-Bayle et al. (25), serum sodium >145 mEq/L; Kahn et al. (24), ≥155 mEq/L*.

*^b^Time when this assessment was done after IVR: in Hanna and Saberi (26) study, within 24 (mean 13.2, SD 5.2) h; in Kahn et al. (24) study, in 24 h; in Sánchez-Bayle et al. (25) study, in 12.34 h (95% CI: 11.94, 12.56)*.

### Risk of Bias Analysis

The assessment of risk of bias is summarized in Table 5 (cohort studies) and Table 6 (RCTs). The mean NOS score of the three cohort studies was 6 (i.e., moderate risk of bias). The criterion that all three studies (24–26) failed to meet, was “comparability,” which requires that cases and controls be matched in the design and/or confounders adjusted for, in the analysis. The main issue was that none of the studies included a control group to compare fluid types, rates, or volumes and thus a risk ratio could not be estimated for any of these factors. Among the three RCTs, one (22) was deemed to be at a very serious risk of bias because the randomization process was not reported, blinding was not performed, and the intention-to treat principle was not applied. The latter is particularly important in view of the fact that 17.7% (22/124) of randomized participants were excluded from the analysis because no blood samples were obtained from them, post IVR treatment.

**Table 5:** Bias assessment for observational studies (Newcastle–Ottawa Scale).

Reference	Design	Selection				Comparability	Outcomes			Total score
		Representativeness of cohort	Selection of non-exposed cohort	Ascertainment of exposure	Outcome of interest	Comparability of cohorts	Assessment of outcome	Adequate duration of follow-up	Adequate follow-up of cohort	
Sánchez-Bayle et al. (25)	Prospective cohort	1	0	1	1	0	1	1	1	6
Kahn et al. (24)	Prospective cohort	1	0	1	1	0	1	1	1	6
Hanna and Saberi (26)	Retrospective cohort	1	0	1	1	0	1	1	1	6

**Table 6:** Bias assessment for randomized control trials.

Reference	Design	Allocation: generation	Allocation: concealment	Blinding	Outcome: complete	Outcome: selective	Other bias
Neville et al. (22)	RCT	Unclear^a^	Low	High^b^	High^c^	Low	Low
Freedman and Geary (21)	RCT	Low	Low	Low	Low	Low	Low
Allen et al. (23)	RCT	Low	Low	Low	Low	Low	Low

*^a^Randomization process not reported*.

*^b^There was no blinding*.

*^c^A significant proportion of subjects (17.7%) was excluded from the final analysis*.

### Narrative Description of Included Studies

Neville et al. conducted a prospective, randomized, non-blinded study that included children with AGE (*n* = 102) comparing the effect of two IVR solution compositions (0.9% saline + 2.5% dextrose vs. 0.45% saline + 2.5% dextrose) on sodium balance (22). The need to administer IVR and the rehydration rate were determined by the attending physician prior to randomization. Rehydration rate options were a “rapid replacement protocol” (RRP; 10 mL/kg/h for 4 h) or a “slow replacement protocol” [SRP; maintenance fluids (27) + estimated dehydration losses as a percentage of body weight replaced over 24 h]. However, the allocated study solution could not be altered during the first 4 h of therapy; after this time window, the attending physicians could adjust IVF management as desired. All included subjects had blood samples drawn prior to the initiation of IVR and again 4 h after the initiation of IVR. Collection of the first passed urine was attempted in all patients. The analysis included 102 children, 37 (36%) of whom were hyponatremic at baseline (serum sodium <135 mEq/L). The authors reported that children who were isonatremic at baseline (serum sodium 135–145 mEq/L) and were treated with a hypotonic IVR solution experienced a mean serum sodium decline of 2.3 (SD 2.2) mEq/L after 4 h of therapy while those who received an isotonic solution experienced only a modest mean increase in serum sodium mean of 0.8 (SD 2.4) mEq/L. Baseline hyponatremia was not corrected after 4 h of hypotonic saline, as the mean serum sodium change in this group was only 0.4 (SD 1.7) mEq/L. Administration of isotonic saline to children with baseline hyponatremia was associated with an increase in mean serum sodium of 2.4 (SD 2.0) mEq/L. The rate of IVR (RRP vs. SRP) was not found to affect serum sodium changes. Forty-two children received IVR beyond 4 h; 22 of these children were initially randomized to the hypotonic solution, and all continued to receive the same solution. Serum sodium values after 24 h of treatment were available in 16/42 children overall and 8 of the 22 in the hypotonic solution arm. Five of the 42 children that received IVR beyond 4 h developed significant hyponatremia (sodium 131 mEq/L) or experienced a serum sodium drop of >4 mEq/L and had a serum sodium value less than 135 mEq/L. All five of these children were among the 22 that received a hypotonic IVR solution beyond 4 h. These findings led the authors to conclude that in children with AGE, IVR with an isotonic solution is preferred since it is more likely to correct baseline hyponatremia and does not appear to be associated with the development of a dysnatremia (low-quality evidence downgraded for serious risk of bias, Tables 6 and 7). These conclusions are limited by the short duration of follow-up biochemistry (4 h) in most of study participants. However, the data from a subgroup of children that were treated beyond 4 h suggest that similar conclusions may apply to IVR continuing for up to 24 h.

**Table 7:** Summary of findings and quality of evidence according to Grades of Recommendation, Assessment, Development, and Evaluation (GRADE) Working Group.

Reference	Outcomes	Narrative results	No. of participants (studies)	Quality of the evidence (GRADE)
Neville et al. (22)	Development of hyponatraemia in children with gastroenteritis receiving IVF of 0.45 vs. 0.9% saline (assessed at 4 h after IVF treatment)	No change in plasma sodium of hyponatremic children receiving 0.45% saline, but a 2.3 (SD 2.2) mmol/L decline in the normonatraemic group. In contrast, 0.9% saline raised plasma sodium by 2.4 (SD 2.0) mmol/L in hyponatraemic children without change in normonatraemic children	102 (1 RCT)	⊕⊕○○ Low^ab^

Freedman and Geary (21)	Development of hyponatraemia in children receiving 60 vs. 20 mL/kg 0.9% saline bolus followed by maintenance 0.9% saline for 3 h (assessed at 4 h after IVF treatment)	A bolus of 60 mL/kg was associated with a greater mean increase in serum sodium of 1.6 (SD 2.4) mEq/L vs. 0.9 (SD 2.2) mEq/L (*P* = 0.04) and was less likely to be associated with a sodium decline of >2 mEq/L (8/112 vs. 17/105; *P* = 0.04) compared with a 20 mL/kg bolus	224 (1 RCT)	⊕⊕⊕⊕ High

Allen et al. (23)	Primary outcome—change in venous serum bicarbonate in dehydrated children with AGE receiving 10–20 mL/kg boluses of 0.9% saline or Plasma-Lyte A for up to 8 hSecondary outcome—shift in serum sodium from baseline (assessed at 4 h after IVF treatment)	Both solutions were associated with correction of baseline hyponatremia in a proportion of children, 4/8 (50%) in the 0.9% saline group and 5/13 (38%) in the Plasma-Lyte A group. In children with normal baseline serum sodium, mild hyponatremia (serum sodium 131–135 mEq/L) developed in one child from each group [1/30 (3.3%) vs. 1/26 (3.9%)]	77 (1 RCT)	⊕⊕⊕⊕ High

Sánchez-Bayle et al. (25)	Development of hyponatraemia in children hospitalized with acute gastroenteritis receiving 0.2–0.3% hypotonic saline (assessed at mean of 12.3 h after IVF treatment)	There were no cases of hyponatraemia post infusion. The mean serum sodium increased by 3.7 mEq/L, decreased by 1.26 mEq/L and decreased by 8.15 mEq/L in patients with hyponatremia, isonatremia, and hypernatremia at baseline, respectively	205 (1 observational study)	⊕○○○ Very low^cd^

Kahn et al. (24)	Neurological complication in children with severe hypernatremic dehydration due to gastroenteritis treated with hypotonic saline (assessed every 6 h for 24 h after IVF treatment)	During the first 24 h, by giving a 70 mEqL saline solution at the rate of 120 mL/kg/24 h, the rate of fall in sodium was below 0.5 mEq/L/h. Rehydration was uneventful in all cases, and no convulsions were observed	40 (1 observational study)	⊕○○○ Very low^cd^

Hanna and Saberi (26)	Incidence and severity of hyponatremia in children with gastroenteritis who had been treated in hospital with hypotonic IVF (0.2–0.45% saline) (assessed at mean of 13.2 h after IVF treatment)	Mean serum sodium declined by 1.7 (SD 4.3) mEq/L in the whole group. Baseline isonatremia was associated with a decline of 1.8 (SD 3.4) to 5.7 (SD 3.1) mEq/L; mild hyponatremia [mean 132.8 (SD 1.3) was associated with an increase of 3.9 (SD 2.5) mEq/L to 136.7 (SD 2.6) mEq/L]	124 (1 observational study)	⊕○○○ Very low^cde^

*GRADE Working Group grades of evidence*.

*High quality: We are very confident that the true effect lies close to that of the estimate of the effect*.

*Moderate quality: We are moderately confident in the effect estimate: the true effect is likely to be close to the estimate of the effect, but there is a possibility that it is substantially different*.

*Low quality: Our confidence in the effect estimate is limited: the true effect may be substantially different from the estimate of the effect*.

*Very low quality: We have very little confidence in the effect estimate: the true effect is likely to be substantially different from the estimate of effect*.

*IVF, intravenous fluid*.

*^a^Was not in adherence with intention-to-treat principle, 17.7% (22/124) participants randomized were excluded from analysis because of not obtaining blood sample post IVF treatment*.

*^b^Not blinding*.

*^c^Lack of a comparison group*.

*^d^No confidence interval estimated*.

*^e^Different outcome time points (4–24 h after IVF treatment)*.

Freedman et al. published results of a randomized blinded trial comparing two rehydration protocols differing in the volume of the initial fluid bolus (60 vs. 20 mL/kg) in 2011 (28). Patients in both study arms received a bolus of 0.9% saline, with the, respectively, allocated volume, followed by maintenance fluids consisting of 5%-dextrose in 0.9% saline at a rate calculated with the Holliday and Segar formula (27). The main outcome measure was achievement of clinical rehydration after 2 h. An analysis of this study’s results focusing on the risk of hyponatremia after 4 h of IVR was subsequently published (21). In total, 224 children, 84 (37%) of whom were hyponatremic at baseline (serum sodium <136 mEq/L), were included. After 4 h of IVR, the proportion of children who were hyponatremic (serum sodium <136 mEq/L) was similar in both arms (21% in the large-bolus group vs. 20% in the standard bolus group, *P* = 0.92). Sixty-three percent (30/48) of children who were hyponatremic at baseline and were treated with large-volume bolus were isonatremic after 4 h of IVR, compared with 44% (15/34) in the standard volume group (*P* = 0.10). Among all participants, irrespective of the bolus size, hyponatremia at baseline was associated with a mean serum sodium increase of 2.6 (SD 2.1) mEq/L compared with only 0.4 (SD 2.1) mEq/L among children who were not hyponatremic (*P* < 0.001). Baseline isonatremic was associated with a plasma sodium decrease of ≥2.0 mEq/L in 17% (23/135) of children, compared with only 2% (2/82) among those who were hyponatremic (*P* = 0.001). Fifty-nine children (53%) in the large-volume bolus group and 39 (37%) in the standard volume group experienced a serum sodium increase ≥2.0 mEq/L, however, no participants developed hypernatremia after 4 h. The conclusion based on these results was that both large and standard volume boluses of 0.9% saline are not associated with development or worsening of hyponatremia after short-term IVR. Moreover, large-volume bolus fluid therapy was associated with a more rapid correction of hyponatremia (high-quality evidence, Table 7). This study’s main limitation is the short duration of follow-up, since serum sodium alterations were only evaluated after 4 h of treatment which does not preclude the development of dysnatremias following more prolonged administration of IVR.

Allen et al. reported the results of a randomized control trial comparing 0.9% saline with Plasma-Lyte A (PLA, Baxter Healthcare, Deerfield, IL, USA) for IVR of children with dehydration caused by AGE (23). The primary outcome of this study was change in serum bicarbonate after 4 h of IVR. The effect on serum sodium was also reported for the subset of participants who had a baseline venous serum bicarbonate ≤22 mEq/L (*n* = 77, including 38 in the 0.9% saline group). Inclusion criteria consisted of AGE in children aged 0.5–11 years with a Gorelick dehydration score ≥4 (29). Prior to randomization participants received standard care, including a 0.9% saline intravenous bolus of up to 20 mL/kg, if considered necessary by the attending physician. Concealed treatment allocation was used with patients being randomly assigned to receive 0.9% saline or PLA in aliquots of 10–20 mL/kg until rehydration was achieved or until the protocol was terminated after 8 h. Maintenance IV fluids were administered if considered appropriate by the attending physician. Blood samples were collected before the first bolus and again 4 h later. While children in the 0.9% saline group were younger (2.9 vs. 3.8 years), baseline mean serum sodium values were similar (136.9 SD 2.93 mEq/L vs. 137.0 SD 4.07 mEq/L). Hyponatremia was defined <135 mEq/L, normal serum sodium 135–145 mEq/L, and hypernatremia as >145 mEq/L. In the 0.9% saline group, eight children had hyponatremia at baseline; four of them had normal serum sodium values 4 h later, the other four remained hyponatremic. In the PLA group, 13 children were hyponatremic at baseline, 5 of whom had normal serum sodium values 4 h later. One patient from each group who had normal serum sodium baseline values developed mild hyponatremia (131–135 mEq/L) after 4 h of IVR. No children were hypernatremic at baseline or follow-up. The authors concluded that the safety profiles of the two tested solutions, with regards to risk for development of dysnatremias, are similar.

Sánchez-Bayle et al. published in 2014 results from a single center prospective observational study that included 205 children aged 1–28 months (mean 0.96, SD 0.48 years) with AGE who received IVR (25). Most study participants (198) were rehydrated with a 0.3% saline + 5% dextrose solution. The hourly rate of IVR was calculated by adding daily maintenance volume according to Holliday and Segar formula (27) to the estimated losses and dividing by 24 h. Only 15/205 (7.3%) of study participants received an isotonic (0.9% saline) fluid bolus at baseline prior to the use of the selected maintenance fluid. Blood samples were collected at baseline and at variable follow-up times thereafter. Analysis included classification of participants according to baseline serum sodium into three groups: hyponatremic (serum sodium <135 mEq/L); normonatremic (serum sodium 135–145 mEq/L); and hypernatremic (serum sodium >145 mEq/L). Urine sample collection was attempted as close to the blood sample as possible. Among all participants, the mean baseline sodium of 141.2 (SD 5.8) mEq/L decreased to a mean of 137.4 (SD 4.1) mEq/L after an average IVR period of 12.34 h (95% CI: 11.94–12.56). Children who were hyponatremic at baseline had an increase in mean serum sodium from 131.9 (SD 2.07) to 135.6 (SD 2.54) mEq/L while isonatremic patients had a modest decrease in mean serum sodium from 139.0 (SD 2.9) to 137.9 (SD 2.5) which was more significant among hypernatremic patients in whom it declined from 150.2 (SD 4.2) to 142.0 (SD 4.3) mEq/L. None of the participants developed or experienced worsening of hyponatremia prior to resuming ORT. The authors concluded that the IVR protocol used in this study based on a hypotonic 0.3% saline + 5% dextrose solution, is safe and effective in children with AGE (very low-quality evidence downgraded for serious risk of bias and imprecision, Table 7). Applicability of this conclusion is limited by the single center design as well as by this cohort’s relatively young average age, 0.96 (SD 0.48) years.

Kahn et al. reported on 40 infants less than 6 months of age who were admitted to the intensive care unit for management of AGE associated hypernatremic dehydration (serum sodium ≥155 mEq/L) (24). Participants were prospectively observed to evaluate the effect of a rehydration protocol that employed a hypotonic rehydration solution containing ~70 mEq/L of NaCl and ~2.5% dextrose that was created by mixing a 145 mEq/L saline solution with 5% dextrose in roughly equal volumes (24). Oral fluids with a solution containing 40 mEq/L of NaCl was allowed when clinically possible, aiming for a total volume of fluid intake (intravenous plus oral) equal to a rate of 120 mL/kg for the first 24 h. Blood samples were collected and weight was measured every 6 h for 24 h. Evaluated outcomes were the slopes of weight increase and sodium decline over time. Results demonstrated that the rate of serum sodium decline was 0.27 (SD 0.04) mEq/L/h; less than the maximum rate of 0.5 mEq/L/h, considered by most experts to be safe (30). Dehydration was slowly corrected as participants gained weight at a rate of 1.74 (SD 0.22) g/kg/h (very low-quality evidence downgraded for serious risk of bias, imprecision, Table 7). The applicability of this study’s results is limited by the specific inclusion criteria targeting young infants with severe hypertonic dehydration as well as by the combination of oral and IVR as 15 of the 40 participants (37.5%) received approximately 50% of their fluids orally.

Hanna and Saberi published a single center retrospective chart review analysis of 124 children, aged 1 month to 12 years, with AGE and dehydration (26). Patients who had their serum sodium measured at baseline and a follow-up performed 4–24 h after initiation of IVR were included in the analysis. Patients with baseline serum sodium <130 or >150 mEq/L were excluded. During the study period, the local rehydration protocol included an optional initial IV bolus of 10–40 mL/kg of 0.9% saline followed by IVR at a rate calculated by adding the volume of clinically estimated losses to maintenance fluids according to the Holliday and Segar formula (27), to be administered over 24 h. The solutions used for IVR during the study period were based on a hypotonic sodium concentration (5% dextrose with 0.2, 0.3, or 0.45% saline). Among 121 children that fulfilled study eligibility criteria, 19 were mildly hyponatremic (serum sodium 130–134 mEq/L), 97 isonatremic (serum sodium 135–145 mEq/L), and 8 mildly hypernatremic (serum sodium 146–150 mEq/L). Results demonstrated that within 24 h of IVR initiation, 18/97 (19%) of children who were isonatremic at baseline developed mild hyponatremia (serum sodium 130–134 mEq/L) associated with an average serum sodium decline of 5.7 (SD 3.1) mEq/L. Compared with children that remained isonatremic, those that developed mild hyponatremia were significantly older (mean 5.8, SD 2.7 years vs. mean 2.8, SD 3.1 years, *P* < 0.0005), more likely to be female (72 vs. 48%, *P* = 0.07) and received a larger volume initial saline bolus (mean 26.1, SD 10.4 vs. mean 20.2, SD 8.6 mL/kg, *P* = 0.06). Among the 19 children that had mild hyponatremia at baseline, 5 remained hyponatremic at follow-up. The overall change in mean serum sodium among all 121 participants was −1.7 (SD 4.3) mEq/L. The main limitation of this study stems from its retrospective design forcing grouping together children that received different IVR hypotonic solutions some of whom received an initial saline bolus of variable volume (very low-quality evidence downgraded for serious risk of bias, inconsistency and imprecision Table 7).

## Discussion

This systematic review confirms the existence of a risk for iatrogenic hyponatremia associated with IVR in children with AGE and dehydration. The risk appears to be more strongly associated with the administration of hypotonic solutions and in certain subpopulations, such as older isonatremic children treated for longer time periods. In addition, this review highlights the lack of a consensus approach to IVR solution composition and rehydration protocols for use in children with AGE. These inconsistencies reflect the limitations of available evidence and the paucity of high-quality articles included in this review. While all included studies examined the effect of IVR on serum sodium and the associated risk of developing a dysnatremia, there were significant differences in the research questions, study designs, and inclusion criteria. Study heterogeneity precluded the conduct of a more sophisticated statistical analysis.

The “maintenance fluids” paradigm, coined by Holliday and Segar in the 1950s, was based on the emulation of physiologic daily requirements for water and electrolytes that need to be administered intravenously, to replace oral intake when it is not possible (27). This approach led to the conception of formulas for calculation of electrolyte quantities and water volumes, that need to be added to calculated deficits and ongoing losses, and are to be administered over 24 h, aiming at meeting requirements, without risk for water or sodium overload. In most cases, such calculations demonstrate that the volume of fluid and quantity of electrolytes needed are approximated best by hypotonic saline solutions containing 0.3–0.45% saline (12). However, despite its sensibility, this approach does not take into consideration non-osmotically induced and occasionally inappropriate excretion of ADH triggered by stress, hypovolemia, and other factors directly related to the AGE itself (31).

Hyponatremia developing in hospitalized children receiving IVFs at appropriately calculated maintenance rates of fluid and electrolytes is well documented (9, 32, 33). Moreover, a higher risk for hyponatremia has been linked to use of hypotonic solutions while isotonic saline was shown to be safe and superior in avoiding hyponatremia in children requiring IVF maintenance (34–38). Nevertheless, many of these studies excluded children with AGE who present unique challenges related to the existence of dehydration, baseline dysnatremias, and ongoing gastrointestinal losses. Evidence available from studies that investigated maintenance IVR in children with conditions other than AGE may not be generalizable to children with AGE. Moreover, the majority of cases reporting potentially fatal iatrogenic hyponatremia associated with hypotonic saline occurred in children with conditions other than AGE in whom the non-osmotic stimulus for ADH secretion may have been stronger and in whom dehydration and ongoing losses were non-existent (39).

This systematic review specifically focused on IVR outcomes in dehydrated children with AGE. While some of the reviewed studies confirmed a significant risk for potentially serious hyponatremia in these children, the review demonstrated that studies comparing isotonic and hypotonic saline are scarce. Only Neville et al. directly investigated this question and their results suggest that in this population, the risk for hyponatremia may be mitigated by using isotonic saline (Table 3). However, risk of bias and GRADE quality assessment of this study suggest that the evidence is of low quality (Table 7). Moreover, the conclusions of this study apply only to relatively short-term IVR protocols, ranging from 4 to a maximum of 24 h. Although the other two RCTs had low risks of bias, the interventions and primary outcomes were not selected to compare risk for dysnatremias between hypotonic vs. isotonic solutions. Freedman et al. used only 0.9% saline to investigate the effect of the rate of rehydration on risk for dysnatremias while Allen et al. focused on correction of venous serum bicarbonate as a primary outcome, comparing two isotonic solutions (21, 23).

The review did not identify sufficient evidence to evaluate the risks and benefits related to different types of IVR solutions among infants less than 1 year of age. The two studies that targeted this population evaluated only hypotonic saline solutions (24, 25). Moreover, Kahn et al. included only infants less than 6 months old with moderate to severe hypernatremia, for which the effective correction requires free water administration which is best achieved with a hypotonic solution (24). In these cases, hypotonic solutions have a clear advantage and should be used to correct hypernatremia; however, the rehydration rate is crucial to maintaining a safe rate of serum sodium correction (40, 41).

In addition to IVR solution tonicity, administered volume, as a function of rate or duration of therapy, can also influence changes in serum sodium. Freedman et al. directly investigated the relationship between the volume of IVR per time unit and risk for dysnatremias using isotonic saline. High-quality evidence (Table 7) generated by this study demonstrated that patients receiving large volumes of isotonic saline were more likely to have mild increases in their serum sodium levels without developing hypernatremia (21). Allen et al. reported similar rates (3–4%) of mild hyponatremia developing after 4 h of IVR with two different isotonic solutions administrated at a relatively standard rate (23). Neville et al., in addition to comparing isotonic with hypotonic saline, analyzed the effect of rehydration rate and they did not identify any evolving cases of hyponatremia after 4 h of therapy with either hypo- or isotonic solution administration (22). However, in a subset of patients who continued with their allocated solution for up to 24 h, 5 of 22 children treated with hypotonic (0.45%) saline developed hyponatremia while none of 20 children that received isotonic (0.9%) saline beyond 4 h did so. This finding highlights the importance of limiting the duration of IVR in children with AGE to the minimum required by promptly resuming ORT as soon as clinically appropriate (42).

This review is limited by the quantity and quality of available evidence. There was significant heterogeneity among included studies with regards to their research question, study design, and baseline clinical characteristics (i.e., age) of the studied populations. Only three short-term clinical trials that focused on different research questions or primary outcomes were identified. Three short-term observational studies analyzed outcome time points that varied from 4 to 24 h. Four included studies were subject to a moderate to high risk of bias (Tables 5 and 6). Weaknesses in study designs, inconsistencies, and imprecisions caused the quality of the evidence generated by four of the included studies to be graded as “low” or “very low” (Table 7). These limitations preclude any conclusive recommendations for IVR in children with AGE and dehydration. Currently, available evidence suggests that isotonic solutions are safe and effective short-term choices for IVR in these children. However, limited evidence also supports the use of hypotonic saline in specific populations such as infants less than 1 year of age. The insufficient data precluded an unbiased comparison between hypotonic and isotonic solutions and thus an optimal IVR solution could not be identified. Thus, future high-quality research investigating outcomes of IVR, utilizing different solutions, in different populations, and for longer periods of time, is required.

## Author Contributions

SG, JX, SS, and SF conceptualized and designed the study, and designed the data collection instruments. SG and JX performed the data extraction. SS and SF supervised and coordinated data collection and analyses. SG drafted the initial manuscript. SG, JX, SS, and SF reviewed and critically revised the drafts of the manuscript and approved the final manuscript as submitted.

## Conflict of Interest Statement

The authors declare that the research was conducted in the absence of any commercial or financial relationships that could be construed as a potential conflict of interest.

## Abbreviations

AGE, acute gastroenteritis; IVR, intravenous rehydration; IVF, intravenous fluids; ORS, oral rehydration solution; CI, confidence interval.
