# Supplementary material for: Iatrogenic Dysnatremias in Children with Acute Gastroenteritis in High-Income Countries: A Systematic Review
Source: Front Pediatr. 2017 Oct 6;5:210. doi: 10.3389/fped.2017.00210 (PMC5635335; doi:10.3389/fped.2017.00210)
Supplement: Supplementary file 1 [file table_1.docx]

**eTable 1.** Search Strategy for Ovid MEDLINE(R) In-Process & Other Non-Indexed Citations and Ovid MEDLINE(R) 1946 to Present.

| ***MeSH and keywords for Gastroenteritis:*** |
| --- |
| 1. exp Gastroenteritis/ |
| 2. Diarrh*ea.mp. |
| 3. exp Vomiting/ |
| 4. or/1-3 [MeSH and keywords for Gastroenteritis] (288751) |
| ***MeSH and keywords for fluid management and dehydration:*** |
| 5. exp Isotonic Solutions/ or exp Hypotonic Solutions/ or exp Infusions, Parenteral/ or exp Fluid Therapy/ or Intravenous fluid.mp. or exp Edema/ or exp Hyponatremia/ |
| 6. exp Blood Volume/ or exp Renin/ or exp Sodium/ or exp Kidney Diseases/ or exp Hypertension/ or exp Kidney/ or exp Blood Pressure/ or Volume expansion.mp. |
| 7. exp Shock, Septic/ or exp Fluid Therapy/ or Fluid management.mp. or exp Pulmonary Edema/ or exp Water-Electrolyte Balance/ |
| 8. exp Dehydration/ or exp Kidney Failure, Chronic/ or exp Acute Kidney Injury/ or exp Rhabdomyolysis/ or exp Kidney/ or exp Fluid Therapy/ or exp Water/ or Hydration.mp. or exp Kidney Diseases/ |
| 9. Dehydration.mp. or exp Dehydration/ |
| 10. exp Hypertension/ or exp Kidney/ or exp Blood Volume/ or exp Blood Pressure/ or Intravascular volume expansion.mp. or exp Hemodynamics/ or exp Heart/ |
| 11. Intravascular volume expansion.mp. |
| 12. Fluid.mp. |
| 13. exp Saline Waters/ or exp Saline Solution, Hypertonic/ or Saline.mp. |
| 14. Rehydration.mp. or exp Fluid Therapy/ |
| 15. or/5-14 [MeSH and keywords for fluid management and dehydration] (2647901) |
| ***MeSH and keywords for hyponatremia and hypernatremia :*** |
| 16. hyponatr*emia.mp. |
| 17. hypernatr*emia.mp. |
| 18. or/16-17 [MeSH and keywords for hyponatremia and hypernatremia] (15175) |
| 19. and/4, 15, 18 (669) |
| 20. limit 19 to humans (602) |
